# An Internet Resource for Self-Assessment of Mental Health and Health Behavior: Development and Implementation of the Self-Assessment Kiosk

**DOI:** 10.2196/mental.9768

**Published:** 2018-05-16

**Authors:** Robert G Maunder, Jonathan J Hunter

**Affiliations:** ^1^ Sinai Health System Department of Psychiatry University of Toronto Toronto, ON Canada

**Keywords:** self-assessment, feedback, surveys and questionnaires, internet

## Abstract

**Background:**

Standardized measurement of physical and mental health is useful for identification of health problems. Personalized feedback of the results can influence health behavior, and treatment outcomes can be improved by monitoring feedback over time. However, few resources are available that are free for users, provide feedback from validated measurement instruments, and measure a wide range of health domains.

**Objective:**

This study aimed to develop an internet self-assessment resource that fills the identified gap and collects data to generate and test hypotheses about health, to test its feasibility, and to describe the characteristics of its users.

**Methods:**

The Self-Assessment Kiosk was built using validated health measurement instruments and implemented on a commercial internet survey platform. Data regarding usage and the characteristics of users were collected over 54 weeks. The rate of accrual of new users, popularity of measurement domains, frequency with which multiple domains were selected for measurement, and characteristics of users who chose particular questionnaires were assessed.

**Results:**

Of the 1435 visits, 441 (30.73%) were visiting for the first time, completed at least 1 measure, indicated that their responses were truthful, and consented to research. Growth in the number of users over time was approximately linear. Users were skewed toward old age and higher income and education. Most (53.9%, 234/434) reported at least 1 medical condition. The median number of questionnaires completed was 5. Internal reliability of most measures was good (Cronbach alpha>.70), with lower reliability for some subscales of coping (self-distraction alpha=.35, venting alpha=.50, acceptance alpha=.51) and personality (agreeableness alpha=.46, openness alpha=.45). The popular questionnaires measured depression (61.0%, 269/441), anxiety (60.5%, 267/441), attachment insecurity (54.2%, 239/441), and coping (46.0%, 203/441). Demographic characteristics somewhat influenced choice of instruments, accounting for <9% of the variance in this choice. Mean depression and anxiety scores were intermediate between previously studied populations with and without mental illness. Modeling to estimate the sample size required to study relationships between variables suggested that the accrual of users required to study the relationship between 3 variables was 2 to 3 times greater than that required to study a single variable.

**Conclusions:**

The value of the Self-Assessment Kiosk to users and the feasibility of providing this resource are supported by the steady accumulation of new users over time. The Self-Assessment Kiosk database can be interrogated to understand the relationships between health variables. Users who select particular instruments tend to have scores that are higher than those found in the general population, indicating that instruments are more likely to be selected when they are salient. Self-selection bias limits generalizability and needs to be taken into account when using the Self-Assessment Kiosk database for research. Ethical issues that were considered in developing and implementing the Self-Assessment Kiosk are discussed.

## Introduction

### Background

Standardized measurement and feedback of aspects of health serves several purposes. Most basically, screening can identify health problems that would benefit from management or treatment. Screening is used for a wide range of health conditions, with variable effects on health outcomes [1-3]. Standardized measurement can also be used to motivate behavior change, for which purpose its effectiveness can be increased by adding personalized feedback about the meaning of scores, including comparison of personal results to population norms. For example, interventions to reduce unhealthy patterns of alcohol consumption in college students are more effective when combined with salient, personalized feedback to enhance motivation for change [4]. Standardized measurement can also serve to assess changes in health phenomena over time. In psychotherapy for mental health problems, for example, routine outcome monitoring with feedback to the therapist (and client) substantially increases the effect size of treatment, reduces dropout rates, and shortens the course of treatment [5]. As another example, cancer patients with metastatic solid tumors who routinely self-assessed common symptoms, with alerts to their oncologist when severe symptoms or worsening were recorded, had significantly increased survival compared with those who did not self-assess symptoms [6].

Many instruments that measure aspects of mental and physical health are available on the internet. These are commonly presented as single-domain measures. It is less common for multiple aspects of health to be measured in the same visit to a single website. Some available internet tools calculate scores automatically (eg, calculators of body mass index or Framingham cardiac risk score), whereas others require scoring by the user. Personalized feedback of scores contextualized with reference to population norms or validated cutoffs for categorical interpretation of scores is uncommon. Thus, commonly available internet tools do not readily serve all of the functions that make standardized health measures valuable.

### Objectives

**Figure 1:**
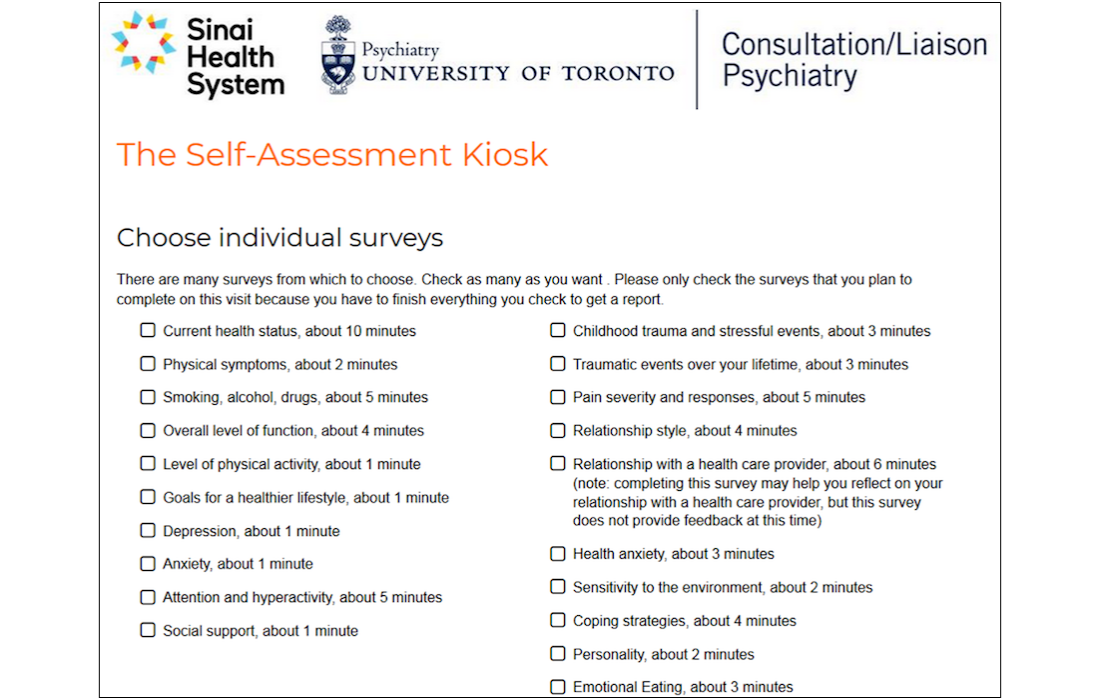
Screenshot of menu page of the Self-Assessment Kiosk.

**Figure 2:**
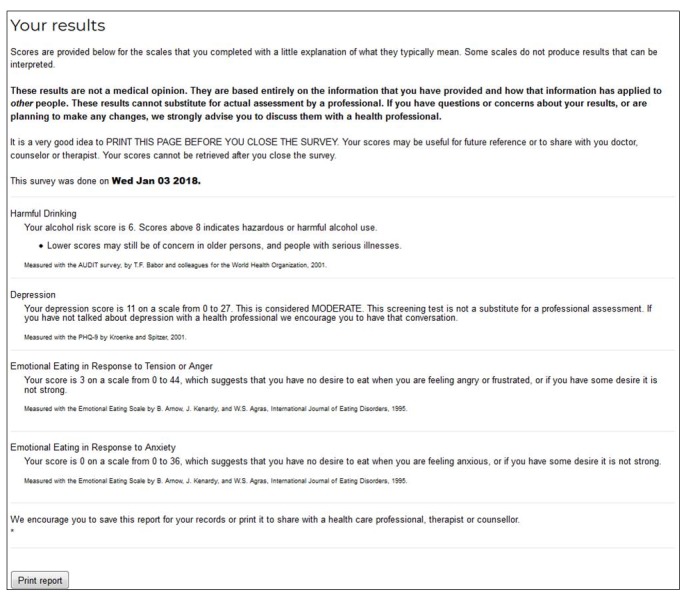
Screenshot of sample results page from the Self-Assessment Kiosk.

Our goal was to develop an internet self-assessment resource that could be used without cost to the user, that would measure a wide range of aspects of mental and physical health with validated instruments, and that would provide both scores and evidence-based feedback about the meaning of those scores to users. We developed the Self-Assessment Kiosk, which provides a menu of health domains from which users can select whichever measures are of interest (Figure 1). After selecting from the menu, the user is then presented with the surveys that have been selected. After completing surveys, the user is provided with scores and feedback that puts the scores into context using established norms or validated cutoffs (Figure 2).

The following necessary characteristics were determined before selecting a platform and instruments to include in the Self-Assessment Kiosk. First, the Self-Assessment Kiosk was intended to be free to users and free of advertising, and so, the costs of implementation and maintenance had to be low. Second, the survey platform used had to be able to score and store scores for many different questionnaires, to be able to score instruments with complex scoring instructions (beyond summing or averaging item scores), and to be able to select text for feedback to users corresponding to the user’s score for each questionnaire. Third, the instruments chosen had to have several characteristics—good evidence for reliability and validity in peer-reviewed journals, published norms or validated cutoffs, to be brief, and to be free. Fourth, permission to use the instrument in the intended context had to be provided by the copyright holder. Adequate safeguards had to be in place to assure the privacy of the information provided.

In addition to serving as a health resource for users, the Self-Assessment Kiosk was also to provide a database for clinical research into the relationships between various aspects of physical and mental health. The authors of the Self-Assessment Kiosk were particularly interested in relationships at the interface between aspects of mental illness, normal psychology, and physical health. As opposed to a hypothesis-driven database comprising measures of constructs specific to a hypothesis, a database that includes many aspects of physical and mental health can serve as a resource that can be interrogated for hypothesis generation or pilot testing of emerging hypotheses over time.

The purposes of this paper are to describe the development and implementation of the Self-Assessment Kiosk and to assess the nature and quality of the data that it collects. Our specific purposes were to test the feasibility of the Self-Assessment Kiosk as a self-assessment resource and as a research tool and to describe the characteristics of its users.

## Methods

### Study Design

The Self-Assessment Kiosk is an open survey. This study of users of the Self-Assessment Kiosk during its first 54 weeks is a cross-sectional study of a convenience sample. The sample consists of users of the Self-Assessment Kiosk who completed at least 1 questionnaire, indicated this was their first visit, consented to research, and indicated that their responses were truthful.

### Development of the Self-Assessment Kiosk

The Self-Assessment Kiosk [7] measures over 20 domains of mental and physical health as well as demographic information and a profile of current medical status. Domains to measure and the instruments to measure them were suggested by the authors (RGM and JJH), with additional input from an advisory committee of the University of Toronto Department of Psychiatry Division of Consultation-Liaison Psychiatry. Permission to use surveys was obtained from the copyright holders. If permission was not granted, alternative instruments were substituted or instruments were excluded. Scoring instructions were obtained from peer-reviewed publications or from the instrument authors.

The Self-Assessment Kiosk was implemented on a commercial internet survey platform (Survey Gizmo, owned by Widgix LLC dba Survey Gizmo, the service provider), which provided the required scoring and skip-logic capabilities to enable adaptive questioning. The service provider implements technical privacy safeguards, which include Secure Sockets Layer linkage and Secure Hash Algorithm-256 with Rivest–Shamir–Adleman-2048 encryption. A service contract stipulates that the service users (authors) retain all rights to the information collected in the surveys, whereas the service provider retains the right to capture the information to use in aggregate (nonidentifying) forms for their own interests. Information is stored in the service provider’s servers “for as long as is needed to provide services to our customers... [and] to comply with [the service provider’s] legal obligations.” Information is downloaded in a database, replaced by an updated cumulative database from time to time, which will be stored on the Sinai Health System server for the duration of the Self-Assessment Kiosk information collection plus 7 years (the latter being a requirement of the Sinai Health System Research Ethics Board, see below).

Legal liability toward users of the Self-Assessment Kiosk was addressed with disclaimers to prevent personalized feedback being interpreted as a medical or diagnostic opinion.

Most instruments selected for the Self-Assessment Kiosk are previously validated [8-28]. These are listed in Table 1 with a summary of instrument characteristics, scoring, and internal reliability (Cronbach alpha). Validated surveys are presented in the Self-Assessment Kiosk with the order of items specified by the instruments’ authors (ie, item order is not randomized).

In addition to validated instruments, questions to assess demographic and descriptive characteristics of users were composed by the authors. To collect information on medical diagnoses (physical and psychiatric), adaptive questioning was used to first screen for any medical condition that requires prescription medication or visits to a health provider more often than routine checkups, and second for any diagnosis within a body system (eg, “High blood pressure, a heart condition, a stroke or a condition of blood vessels.”). Users who endorse a diagnosis within a body system are then provided a list of the most common diagnoses within that system plus an “other” (write-in) option. In this way, a large number of diagnostic conditions (84 plus “other”) could be screened with a smaller number (17) of mandatory screening questions.

All users also complete measures of physical and emotional well-being at the time of the survey (visual analog scales from 0 to 100) and a seriousness check, which has been shown to increase the validity of Web-based self-reports [29]. The seriousness check used was the following: “Sometimes people ‘check out’ the Self-Assessment Kiosk without providing information that is true about them. That is fine, but we would like to know. Have you answered the questions in the surveys you completed today honestly, based on your current circumstances?”

A summary page provides scores and places these scores in context with brief text that is based on existing norms or validated cutoffs. Text for feedback to users based on instrument scores was written by the authors and presented to users via a combination of the survey platform’s built-in user-feedback functions and scripts written in JavaScript and embedded in the survey. The Self-Assessment Kiosk was field tested by authors and volunteers until no bugs were detected in several consecutive usages before launch.

### The Self-Assessment Kiosk User Experience

Users of the Self-Assessment Kiosk are encouraged to use the resource as often as they wish. Therefore, at the start of any visit, users are asked if this is a first visit or a return visit. Users are also presented the option of providing a username, which allows information from different sessions to be linked by the authors. The user experience is identical whether they provide a username or not.

On the first visit (and at any subsequent visit if desired), users are provided information about the Self-Assessment Kiosk and presented with a research consent page. Users are informed that their experience is identical whether they agree that their information may be used for research purposes or not. The consent page informs users who the investigators are (RGM and JJH), the purpose of research (“to understand how aspects of psychological health and physical health are related to each other”), the length of the survey (“as little as 5 minutes or… over an hour, depending on how many surveys you decide to do”), and that aggregate results of this research, for those who consent, may be published in a medical or scientific journal or may be presented at a medical or scientific meeting. Users are informed about anonymity (“using the Self-Assessment Kiosk is anonymous… we do not ask for your name or your email address and we do not record information about your computer”) and limitations on privacy protections (“however, the surveys do ask many personal questions, including questions about medical conditions, mental health conditions, age, gender, what country you live in, your ethnicity, and questions about traumatic or stressful life experiences.

**Table 1:** Domains measured and characteristics of instruments used in the Self-Assessment Kiosk.

Domain	Instrument	Summary of characteristics
Impact of illness	Illness Intrusiveness Rating Scale [8]	13 items rated 1 to 7; scale score is item sum; alpha=.94
Anxiety	GAD^a^-7 [9]	7 items rated on 4-point scale from 0 to 3; scale score is item sum; alpha=.91
Depression	PHQ^b^-9 [10]	9 items rated on 4-point scale from 0 to 3; scale score is item sum; alpha=.90
Physical symptoms	PHQ^b^-15 [11]	15 items rated on 3-point scale from 0 to 2; scale score is item sum; alpha=.81
Smoking	CAMH^c^ monitor survey [12]	4 items probing current and past smoking, duration and amount; no summary score; internal reliability not applicable
Alcohol use problems	Alcohol use disorder identification test [13]	8 items rated on a 5-point scale from 0 to 4, 2 items rated on a 3-point scale as 0, 2, and 4; scale score is item sum (items 4 to 8 are skipped and scored 0 if sum of items 1 to 3=0); alpha=.85
Disability	Brief WHO^d^ Disability Assessment Scale, WHODAS 2.0 [14]	12 items measuring function in different domains, each rated from 1 to 5; scale score is item mean; alpha=.93; plus 3 items probing number of disabled days in past month
Physical activity	Stanford brief activity survey [15]	2 items probing typical on-the-job activity and leisure activity, each with 5 possible response categories representing increasing intensity of activity. Scoring by categorization into 5 categories of overall activity, based on a 5×5 scoring grid; internal reliability not applicable
ADHD^e^	ADHD self-report scale [16]	18 items answered on a 5-point response scale which is then dichotomized (0 or 1) for each item. Outcome is dichotomous: low probability of ADHD (score of 0-3 on first 6 items) or high probability of ADHD (score of 4-6 on first 6 items); alpha (6-item)=.68, alpha (18-item)=.86
Social support	ENRICHD^f^ social support inventory [17]	6 items scored from 1 to 6; scale score is item sum; alpha=.92
Childhood adversity	Adverse Childhood Experience (ACE) Survey [18]	17 items answered yes or no to score 10 categories of types of adversity (0 or 1); summed to yield ACE score (0-10). Internal reliability not applicable
Lifetime trauma	Brief trauma questionnaire [19]	9 items used to survey the occurrence of types of traumatic exposure (yes or no); endorsed items were followed with 2 severity questions (yes or no); this yielded dichotomous assessment of exposure to traumatic experiences which led to serious injury or were perceived to threaten life or serious injury (yes or no); internal reliability was not applicable
Appraisal of pain	McGill pain questionnaire [20]	20 types of pain descriptor adjectives, 1 temporal pattern item, 6 types of pain severity adjectives, with adjectives ordered for scoring within categories, and categories varying from 3 to 6 possible responses (or 0 if left blank). Score is item sum (0-78); alpha=.78
Catastrophizing	Pain catastrophizing scale [21]	13 items scored 0 to 4; score is item sum; alpha=.95
Attachment insecurity	Experience in close Relationships-M16 [22]	16 items scored from 1 to 7, 8 items for attachment anxiety, 8 items for attachment avoidance (3 items are reverse scored); scores are item means; attachment anxiety alpha=.86, attachment avoidance alpha=.86
Treatment alliance	Human connection scale [23]	16 items scored 1 to 4; score is item sum; alpha=.92
Health anxiety	Health anxiety inventory [24]	18 items, scored on a 4-point scale from 0 to 3; score is item sum; alpha=.91
Sensitivity to Environment	Highly sensitive person scale [25]	11 items scored from 1 to 7; score is item mean; alpha=.94
Coping	Brief COPE [26]	28 items scored 1 to 4; 14 methods of coping scored as the mean of 2 items; alpha values: self-distraction=.35, active coping=.76, denial=.68, substance use=.96, emotional support=.84, instrumental support=.83, behavioral disengagement=.66, venting=.51, positive reframing=.83, planning=.81, humor=.81, acceptance=.50, religion=.83, and self-blame=.82
Personality	Ten-item personality inventory [28]	10 items scored on a 7-point scale from 1 to 7; mean of 2 items (1 reverse scored) for each of 5 personality domains; alpha values: extraversion=.71, agreeableness=.46, conscientiousness=.69, neuroticism=.72, and openness=.45
Emotional eating	Emotional eating scale [28]	25 items scored on a 5-point scale (0-4); 11 items are summed for eating in response to anger (0-44) and 9 items are summed for eating in response to anxiety (0-36); alpha values: anger=.90 and anxiety=.85

^a^GAD: generalized anxiety disorder.

^b^PHQ: Patient Health Questionnaire.

^c^CAMH: Centre for Addiction and Mental Health.

^d^WHO: World Health Organization.

^e^ADHD: attention deficit and hyperactivity disorder.

^f^ENRICHD: Enhancing Recovery in Coronary Heart Disease.

The information that you provide will be stored within the survey system databases and downloaded to be stored on servers at Mount Sinai Hospital in Toronto, Canada... Information that travels on the internet and is stored on a computer is sometimes viewed by third parties by accident, because of government policies or a court subpoena, as a result of criminal hacking or for other reasons. Therefore, using the Self-Assessment Kiosk carries some risk of your personal, anonymous information being read by someone other than the researchers.”). Note that although there are technical safeguards against a privacy breach, the consent process emphasizes sources of risk and the primary safeguard of maximizing the anonymity of data.

Users are warned about risks of using the Self-Assessment Kiosk, which exist whether or not they consent to research. These are privacy risks (as above), and the risk that responding to surveys about mental health and trauma may cause distress or trigger bad memories. They are also informed of safeguards against this distress (“You will only see these surveys if you ask to. You are allowed to skip questions if you don’t want to answer them. Surveys that ask about traumatic experiences are preceded by a trigger warning that allows you skip them if you want to.”).

Users are also informed about the limitations on the validity of feedback (“Feedback puts your results in context, based on what similar results have meant for other people in research studies. The feedback is not about you personally, because completing surveys is not the same as a medical or psychological assessment. As a result, the feedback may or may not be accurate for you. Reading feedback about your answers to psychological or medical questions may raise concerns or questions for you that the providers of the Self-Assessment Kiosk are unable to answer or respond to. We encourage you to discuss these concerns and questions with a health professional.”). The process of providing information and obtaining consent has been approved by the Mount Sinai Hospital Research Ethics Board (REB #16-0186).

After choosing to consent to research or not, users choose individual domains to assess from a menu and are also offered bundles of preselected combinations of survey instruments, including the option of a comprehensive assessment (all instruments). The approximate time required for each instrument is provided in the selection menu (see Figure 1). To maximize anonymity and minimize distress, almost every item within the Self-Assessment Kiosk can be skipped if desired. Demographic and other descriptive characteristics of users are collected for users who are visiting the Self-Assessment Kiosk for the first time and not for users who indicate they have visited previously.

As users can choose a large number of questionnaires, steps were taken to reduce fatigue. The presentation of survey items varies to maintain user interest, including check boxes, short write-in answers, radio buttons, and sliders. For users who select a large number of surveys, brief “fun fact” quizzes with immediate feedback about responses are interspersed between some surveys to provide variety and interrupt fatigue. The number of items per screen is generally less than 10 and varies from 1 to 15. Users can navigate with back buttons to revise answers if they wish.

After completing the selected surveys, users are provided with personalized feedback based on published norms and cutoffs on a summary page that can be saved as a file or printed for future reference. Feedback for scores that indicate a possible need for professional assessment or treatment includes the suggestion to discuss the results with a physician.

### Distribution of the Self-Assessment Kiosk

The target population of the Self-Assessment Kiosk is broad, consisting of any interested adult with access to the resource. It was marketed to potential users in various ways (Multimedia Appendix 1). Two email distribution notices were sent to primary care providers, psychotherapists and psychiatrists known to the authors, first in Sept 2016 and then in January 2017. Periodic notices were posted on a Twitter account that had approximately 1000 followers during the period of this study (@boiby). In each case, readers of the notice were encouraged to distribute it widely. Health-related Web resources were contacted to request that a link to the Self-Assessment Kiosk be included on websites. As a result of this snowballing method of distribution of the link to the survey, it is not known what websites or other internet sources provide access to the Self-Assessment Kiosk.

### Statistical Analysis

Descriptive data were tabulated to characterize users and their usage of the Self-Assessment Kiosk over its first 54 weeks after launch. In addition to the demographic characteristics of users, we calculated the rate of accrual of new users and the median number of questionnaires that are usually completed.

The popularity of each measurement domain was determined by calculating the rate at which each questionnaire was completed. To demonstrate the reduction in available sample size when the relationship between multiple questionnaires is studied, we calculated the rate of completion of a combination of 3 questionnaires, then repeated this for a different combination of 3 questionnaires.

The influence of personal characteristics on the choice of which questionnaires to complete was tested with logistic regression entering the binary variable (completed or not completed) as dependent variable and age, gender, medical diagnosis (any or none), education, and income category as independent variables. Questionnaires that were completed by at least 20% of the user cohort were included in this analysis. Data were analyzed in IBM SPSS Statistics for Windows Version 24.0 (IBM Corporation, Armonk, New York).

## Results

### Description of the Cohort

**Figure 3:**
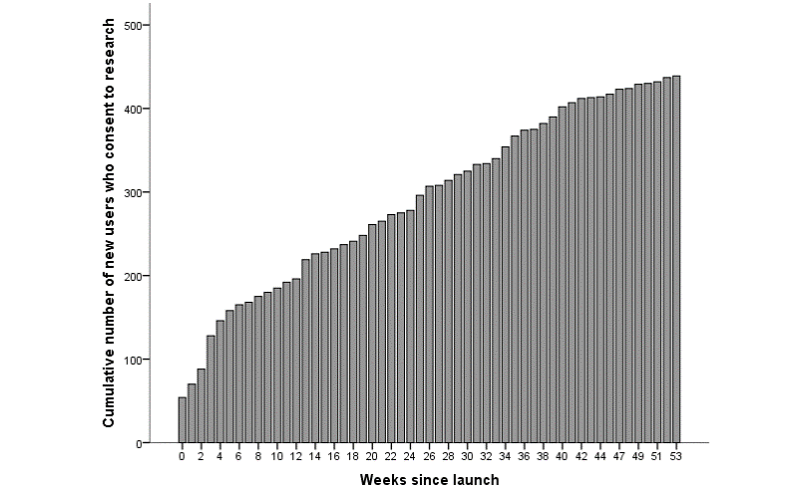
Accumulation of new users of Self-Assessment Kiosk who consent to research.

In the first 54 weeks after its launch, the Self-Assessment Kiosk received 1435 visits. Of these, 762 visits included completion of at least 1 questionnaire, 601 for the first time and 161 as return users. The number of visits grew each week, by a median of 14 users per week (interquartile range 3-34). Of 601 first-time users who completed questionnaires, 73.4% (443/601) provided consent for the use of their information for research purposes. Of these, in answer to the question about the seriousness of their responses, 2 users indicated that their answers were not entirely truthful. This report concerns the remaining 441 people. Growth in the number of users over time was approximately linear after the first few weeks, as illustrated in Figure 3.

Descriptive characteristics of the cohort are presented in Table 2. The modal user was a highly educated, married, white woman with an income greater than $75,000 annually. Most users were from Canada or the United States. Of users who provided information about medical diagnoses, more than half (53.9%, 234/434) reported at least 1 medical condition. The median number of questionnaires completed on the first visit was 5 (interquartile range 2-10).

### Relationship of User Characteristics to Questionnaire Selection

As indicated in Table 3, the most commonly chosen questionnaires measured depression (61.0%, 269/441), anxiety (60.5%, 267/441), attachment insecurity (54.2%, 239/441), and coping (46.0%, 203/441). Users’ demographic characteristics were associated with the choice to complete or not complete most measures, although these characteristics explained a small portion of the variance in this choice (*R*^2^<.09; Table 3). The most consistent trend was a lower tendency to complete measures in users with higher income.

The mean Patient Health Questionnaire-9 (PHQ-9) score of 269 Kiosk users who completed the measure of depression (mean 8.4, SD 6.3) was intermediate between previously studied populations [10] with major depressive disorder (mean 17.1, SD 6.1), other depressive disorder (mean 10.4, SD 5.4), or no depressive disorder (mean 3.3, SD 3.8). The distribution of PHQ-9 score of Kiosk users compared with these previous cohorts is shown in Table 4.

The mean score of 267 Kiosk users who completed a measure of anxiety (GAD-7) was 7.2 (SD 5.1), which was intermediate between patients with generalized anxiety disorder (mean 14.4, SD 4.7) or no generalized anxiety disorder (mean 4.9, SD 4.8) in a previously reported study [9]. In this study, the distribution of GAD-7 scores was minimal (0-4): 37.8% (101/267), mild (5-9): 39.7% (82/267), moderate (10-14): 22.5% (60/267), and severe (15-21): 9.0% (24/267).

**Table 2:** Characteristics of 441 users of Self-Assessment Kiosk.

Characteristic		n (%)^a^
**Gender (N=437)**		
	Female	336 (76.2)
	Male	89 (20.2)
	Other	12 (2.7)
**Age in years (N=433)**		
	Under 30	86 (19.5)
	30-50	198 (44.9)
	51-65	110 (24.9)
	Over 65	39 (8.8)
**Education (N=433)**		
	High school or less	22 (5.0)
	Some college, no degree or diploma	39 (8.8)
	College diploma or bachelor’s degree	132 (29.9)
	Graduate or professional degree	240 (54.4)
**Income (N=383)**		
	<$35,000	68 (15.4)
	$35-75,000	89 (20.2)
	$75-150,000	120 (27.2)
	>$150,000	106 (24.0)
**Relationship status (N=427)**		
	Single	127 (28.9)
	Married or common-law	236 (53.5)
	Separated or divorced	56 (12.7)
	Widowed	8 (1.8)
**Country of birth (N=427)**		
	Canada	367 (83.2)
	United States	44 (10.0)
	Other	16 (3.7)
**Race or ethnicity (N=426)**		
	Asian	40 (9.1)
	Black or African origin	11 (2.5)
	White or European origin	336 (76.5)
	Hispanic or Latino	6 (1.4)
	Aboriginal or Native origin	2 (0.5)
	Other	31 (7.1)

^a^Percentage of entire sample (N=441) adds up to less than 100% because of missing data.

**Table 3:** Relationship of user characteristics with choice to complete questionnaires or not.

Dependent variable (questionnaire completed)	n (%)	Odds ratios					Model	
		Gender	Medical diagnosis	Age	Education	Income	*R* ^2^	*P* value
Depression^a^	269 (61.0)	1.5	0.9	0.9	0.9	0.6^b^	.08	<.001
Anxiety^c^	267 (60.5)	1.7^d^	1.0	0.8	0.8	0.8^d^	.06	<.001
Attachment insecurity^e^	239 (54.2)	1.2	0.7	0.8	1.0	0.7^f^	.04	.006
Coping^g^	203 (46.0)	1.5	0.8	0.9	1.0	0.7^f^	.05	.003
Physical activity	177 (40.1)	1.0	0.9	1.1	0.8	0.9	.01	.55
Social support^h^	172 (39.0)	1.3	1.1	1.0	1.0	0.7^b^	.05	.001
Physical symptoms^i^	165 (37.4)	1.0	0.9	1.0	0.7^d^	0.8^d^	.04	.01
Sensitivity to environment^j^	162 (36.9)	1.8^d^	0.7	0.8	0.8	0.8	.05	.004
Stage of change	162 (36.7)	1.4	1.1	1.1	0.9	0.8	.02	.19
Health anxiety	156 (35.4)	1.4	0.8	0.9	0.8	0.9	.02	.15
Lifetime trauma^k^	148 (33.6)	1.3	1.2	0.9	1.1	0.6^b^	.07	<.001
Childhood adversity^l^	144 (32.7)	1.2	1.1	0.8	1.0	0.6^b^	.06	<.001
Alcohol use disorder	144 (32.7)	0.9	1.2	0.9	0.8	0.8	.03	.04
Attention deficit^m^	144 (32.7)	1.3	1.1	0.8	0.8	0.6^b^	.08	<.001
Overall function^n^	131 (29.7)	1.0	1.1	0.7^d^	0.7^d^	0.8	.05	.002

^a^Completion of depression measure associated with lower income.

^b^*P*<.001.

^c^Completion of anxiety measure associated with female gender and lower income.

^d^*P*<.05.

^e^Completion of attachment measure associated with lower income.

^f^*P*<.01.

^g^Completion of coping measure associated with lower income.

^h^Completion of social support measure associated with lower income.

^i^Completion of physical symptom measure associated with lower education and lower income.

^j^Completion of sensitivity to environment measure associated with female gender.

^k^Completion of lifetime trauma measure associated with lower income.

^l^Completion of childhood adversity measure associated with lower income.

^m^Completion of attention deficit measure associated with lower income.

^n^Completion of overall function measure associated with lower education and younger age.

**Table 4:** Distribution of depression scores (PHQ-9) in Self-Assessment Kiosk users compared with other cohorts.

Severity	Self-Assessment Kiosk (N=269), n (%)	Comparison cohorts (from Kroenke [10]), n (%)		
		Major depression (N=41)	Other depressive disorder (N=65)	No depressive disorder (N=474)
Minimal (0-4)	89 (33.1)	1 (2)	8 (12)	348 (73.4)
Mild (5-9)	86 (19.6)	4 (10)	23 (35)	93 (19.6)
Moderate (10-14)	42 (15.6)	8 (20)	17 (26)	23 (4.9)
Moderately severe (15-19)	34 (12.6)	14 (34)	14 (22)	8 (1.7)
Severe (20-27)	18 (4.1)	14 (34)	3 (5)	2 (0.4)

### Proportion of Users Who Complete Multiple Questionnaires

Future studies of relationships between variables in the Kiosk will require studying responses from users who have completed questionnaires that measure each of the constructs involved in the relationship. In general, the more questionnaires that are involved in a relationship, the fewer the number of users who have provided complete information. To explore how large these user subsets might be, and thus how many users must accrue to study multivariable relationships, we calculated the available sample for 2 examples: (1) a study of a hypothesis regarding a relationship between depression, childhood adversity, and health anxiety and (2) a study of a hypothesis regarding a relationship between physical symptoms, harmful alcohol consumption, and attention deficit symptoms.

For the first example, of the 439 users, 61.3% (269/439) completed a measure of depression; of those, 27.8% (122/439) also completed a measure of childhood adversity; and of those, 22.1% (97/439) also completed a measure of health anxiety. For the second example, of the 439 users, 37.6% (165/439) completed a measure of physical symptoms; of those, 25.1% (110/439) also completed a measure of harmful drinking; and of those, 21.9% (96/439) also completed a measure of attention deficit. Thus, in these examples, a moderately complex hypothesis (3 variables) requires up to 2- to 3-fold greater participant accrual for a given sample size requirement than a single-variable study.

## Discussion

### Feasibility

The Self-Assessment Kiosk is a free resource available to the general public. Modest efforts at marketing resulted in a steady accumulation of new users over its first year of availability, such that the rate of new users was close to linear (Figure 1). The consistency of the accumulation of new users suggests that it is feasible to expect continued use of the resource over time.

Users of the Self-Assessment Kiosk tended to complete multiple questionnaires (median 5). Completion of multiple questionnaires allows the Self-Assessment Kiosk database to be interrogated to understand the relationships between variables, for purposes such as hypothesis generation. As users choose whatever combination of surveys that they prefer, assessing relationships between multiple instruments reduces the available sample size of users who have completed all relevant measures. However, 2 examples of 3-variable relationships that we tested indicate that this is a surmountable challenge, in that a 3-variable hypothesis requires just 2 to 3 times the size of Self-Assessment Kiosk users as a 1-variable hypothesis. The availability of sufficient users to test more complex hypotheses essentially depends on the rate of accumulation of new users, which in turn depends on time and marketing effectiveness.

### Characteristics of Users

Analysis of the range of scores of users of the PHQ-9 and GAD-7 indicates that users who select particular instruments tend to have scores that indicate the presence of some symptoms, although these are less severe, on average, than those found in a clinical cohort. This indicates that users tend to choose instruments that are salient to them. With respect to using the Self-Assessment Kiosk database as a research resource, this introduces selection biases and needs to be taken into account when interpreting results.

### Reliability and Generalizability

The internal reliability of the measures used in the Self-Assessment Kiosk indicates that most instruments have retained the reliability that has been established in pen-and-paper versions when used in this new context. Lower internal reliability of 3 subscales of the coping instrument and 2 subscales of the personality instrument suggest the need for cautious interpretation of the results of these measures and ongoing reassessment of whether alternative measures of these constructs would be more robust for an open internet survey.

There are limits on the generalizability of results of analyses of data from the Self-Assessment Kiosk. Compared with the general population, users of the Kiosk were biased toward female gender, high income and education, and ages between 30 and 50 years. These demographic biases also make the Self-Assessment Kiosk a more appropriate research tool for hypothesis generation than for hypothesis testing. It is noteworthy that greater demographic representativeness may be possible with marketing that targets distribution to a wider population. Additionally, when the number of users is sufficiently large, users can be purposively sampled and stratified to create more representative cohorts.

### Ethical Issues

Ethical issues regarding the potential for harm were carefully considered before releasing the Self-Assessment Kiosk, including review by the Research Ethics Board of our institution. Related concerns about perceived legal liability were also considered and discussed with legal counsel. Issues considered and the remedies that were applied included the following. First, we considered the potential for harm when sensitive psychological information is provided to people without knowledge of their access to health and mental health services. This concern is balanced against the potential value of putting information about mental and physical health (which, by definition, is already known to a person who completes a self-report measure) into the context of established norms and correlations, which may provide either reassurance or motivation to seek treatment. Second, we specifically considered the related risk that asking about experiences of trauma could be retraumatizing or “triggering” in some circumstances. This concern was dealt with through informed consent, by providing explicit trigger warnings with options to skip trauma questionnaires (even after choosing these questionnaires in the menu) and allowing users to skip individual questions. Third, we considered the professional ethical imperative not to provide diagnostic assessment for people in the absence of psychiatric or psychological assessment. Diagnosis is not possible in the absence of professional evaluation and is not the intent of the Kiosk. This limit is clearly stated in disclaimer messages provided before collecting any information from Kiosk users and repeated when feedback is provided. The author of 1 instrument declined permission to use that instrument based on the concern that measurement in the absence of a professional relationship was deemed to fall short of professional ethical requirements, indicating that there is a range of opinions about this choice. Fourth, we considered the potential for harm as a result of an information breach of a database containing personal health information. This concern was dealt with by designing the Kiosk to provide all of its functions to users who remain anonymous and through informed consent with respect to the residual risk.

### Summary

The Self-Assessment Kiosk demonstrates the feasibility of a low-cost, automated internet resource to provide a personalized menu of valid psychological measures with feedback to users. The data that users provide can serve as a rich source of investigation for the purposes of generating hypotheses and pilot data.

## Appendix

Multimedia Appendix 1 Advertising of the Self-Assessment Kiosk.

## Abbreviations

ADHD: attention deficit and hyperactivity disorder
CAMH: Centre for Addiction and Mental Health
ENRICHD: Enhancing Recovery in Coronary Heart Disease
GAD: generalized anxiety disorder
PHQ: Patient Health Questionnaire
WHO: World Health Organization
