# Supplementary material for: An Internet Resource for Self-Assessment of Mental Health and Health Behavior: Development and Implementation of the Self-Assessment Kiosk
Source: JMIR Ment Health. 2018 May 16;5(2):e39. doi: 10.2196/mental.9768 (PMC5981055; doi:10.2196/mental.9768)

A. Example of email sent to colleagues

Re: A self-assessment resource for patients and for others

Dear friends and colleagues,

We have launched an online resource that provides validated surveys that are freely accessible to all, and provides scores and feedback. **The Self-Assessment Kiosk** is a source of questionnaires that measure a wide range of constructs that are relevant to physical and mental health (and particularly to health psychology).

The Self-Assessment Kiosk is a very user-friendly and flexible resource that allows users to choose what they want to measure and provides feedback based on published norms and validated cut-offs whenever these are available. Users can choose to save a report with their results or to print the report. Thus, if you wish, patients can share the results of measures with you and follow their progress over time. We have attached a “requisition” form that you may want to use if it helps to facilitate your patients sharing self-assessment results with you.

Users have the option of choosing to consent to their results being used in research or not, as they wish. We envision a growing database of consenting users whose survey results can be used to generate new hypotheses about the relationships between physical and psychological phenomena.

We are asking for your help in getting our invitation out to the public. Anyone who has access to the Internet and speaks English is able to complete surveys and is welcome to participate. The research portion of the Self-Assessment Kiosk project has been approved by the Mount Sinai Hospital Research Ethics Board.

Please forward this email to your other contacts, as widely as you wish, or post the attached poster wherever members of the public will have access to it.

And have a look yourself; the easiest way to see what the Self-Assessment Kiosk can do is to give it a test run. The survey is available at this link: **http://bit.ly/2bAz2NP**

B. Example of flyer/poster distributed to colleagues to post wherever they wish and posted on the website www.attachmentandhealth.com.

Sinai Health System

Psychiatry UNIVERSITY OF TORONTO

Consultation/Liaison Psychiatry

The Self-Assessment Kiosk

Assess your own physical and emotional health

The Self-Assessment Kiosk is a free online source of validated questionnaires which assess many aspects physical and mental health.

The Self-Assessment Kiosk is a very user-friendly and flexible resource that allows you to choose what you want to measure and provides feedback about your scores.

All information that you provide to the Self-Assessment Kiosk is completely anonymous. You can choose to save or print a report of your results. If you wish, you can share your results with a doctor or health care provider.

You also can choose to consent to your results being used in research or not, as you wish. Over time, we will use the results of research on the Self-Assessment Kiosk to generate new hypotheses about how physical and mental health are related. The research portion of the Self-Assessment Kiosk has been approved by the Mount Sinai Hospital Research Ethics Board.

The Self-Assessment Kiosk can be found at http://bit.ly/2ccH0tx

or by following this square code

C. Typical marketing tweet

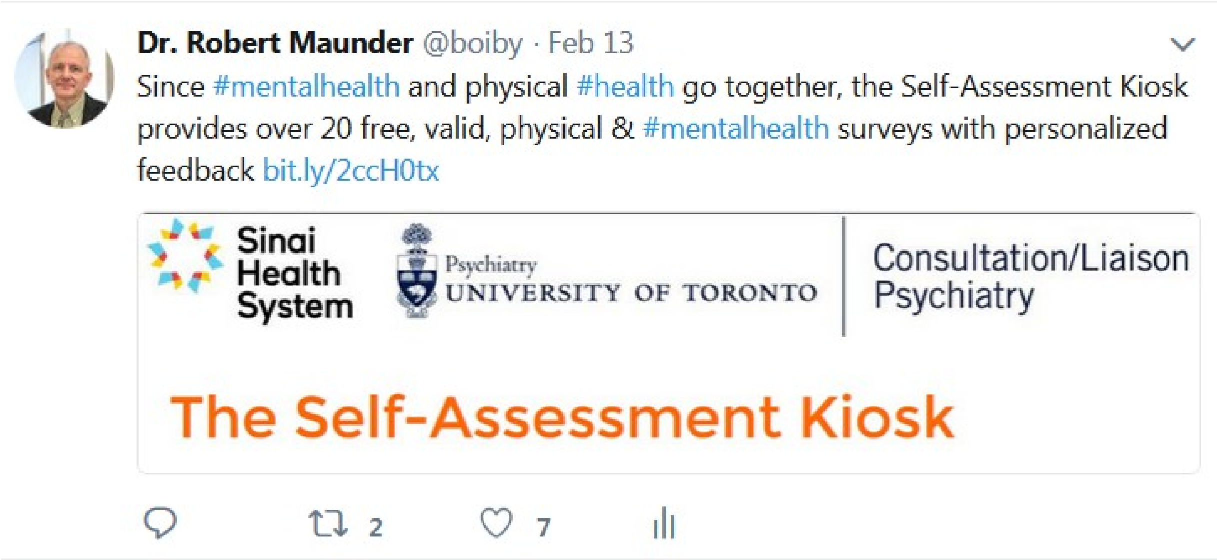

Supplement: Multimedia Appendix 1 [file mental_v5i2e39_app1.pdf]
